# Expansions of Cytotoxic CD4^+^CD28^−^ T Cells Drive Excess Cardiovascular Mortality in Rheumatoid Arthritis and Other Chronic Inflammatory Conditions and Are Triggered by CMV Infection

**DOI:** 10.3389/fimmu.2017.00195

**Published:** 2017-03-02

**Authors:** Iain Broadley, Alejandra Pera, George Morrow, Kevin A. Davies, Florian Kern

**Affiliations:** ^1^Division of Medicine, Brighton and Sussex Medical School, Brighton, UK; ^2^Department of Immunology, Maimonides Institute for Biomedical Research (IMIBIC), Reina Sofía University Hospital, University of Cordoba, Cordoba, Spain

**Keywords:** CD4 T cells, cytotoxic T cells, cardiovascular diseases, chronic inflammatory disease, autoimmune diseases

## Abstract

A large proportion of cardiovascular (CV) pathology results from immune-mediated damage, including systemic inflammation and cellular proliferation, which cause a narrowing of the blood vessels. Expansions of cytotoxic CD4^+^ T cells characterized by loss of CD28 (“CD4^+^CD28^−^ T cells” or “CD4^+^CD28^null^ cells”) are closely associated with cardiovascular disease (CVD), in particular coronary artery damage. Direct involvement of these cells in damaging the vasculature has been demonstrated repeatedly. Moreover, CD4^+^CD28^−^ T cells are significantly increased in rheumatoid arthritis (RA) and other autoimmune conditions. It is striking that expansions of this subset beyond 1–2% occur exclusively in CMV-infected people. CMV infection itself is known to increase the severity of autoimmune diseases, in particular RA and has also been linked to increased vascular pathology. A review of the recent literature on immunological changes in CVD, RA, and CMV infection provides strong evidence that expansions of cytotoxic CD4^+^CD28^−^ T cells in RA and other chronic inflammatory conditions are limited to CMV-infected patients and driven by CMV infection. They are likely to be responsible for the excess CV mortality observed in these situations. The CD4^+^CD28^−^ phenotype convincingly links CMV infection to CV mortality based on a direct cellular-pathological mechanism rather than epidemiological association.

## Introduction

CD28 is a costimulatory molecule expressed on naïve CD4^+^ and CD8^+^ T cells. A permanent loss of CD28 occurs during antigen-driven differentiation toward a terminal phenotype. Its loss suggests that costimulation by antigen-presenting cells (APC) *via* its specific ligands B7.1 (CD80) and B7.2 (CD86) is no longer required and is indicative of replicative senescence (1). This should not be confused with the transient loss of CD28 expression on CD4^+^ (and CD8^+^) T cells during proliferation, which is reversible within days (1).

CD4^+^CD28^−^ T cells were first identified in the plaques of patients with unstable angina but since then, expansions of these cells have been reported in a range of cardiovascular (CV) conditions. They attracted particular interest in acute coronary syndrome (ACS) and myocardial infarction where their presence was associated with increased acute mortality and recurrence (2–4). Patients with CD4^+^CD28^−^ T cell expansions also showed preclinical atherosclerotic changes (5). A recent study of ACS with/without diabetes mellitus (DM) reported the highest frequencies of CD4^+^CD28^−^ T cells when both conditions were present, followed by ACS only, DM only, and finally controls (6).

As regards autoimmune diseases, expansions of so-called “CD4^+^CD28^null^” (synonymous for CD4^+^CD28^−^) were described in rheumatoid arthritis (RA) patients almost 20 years ago (7). Their limited TCR Vβ chain usage suggested restricted antigen-specificity and potential involvement in autoimmunity; interestingly, their numbers were related to the extent of extra-articular involvement (7–9). Over the years, CD4^+^CD28^−^ T cells have been shown to be implicated in various inflammatory conditions (10) including granulomatosis with polyangiitis (GPA), where CD4^+^CD28^−^ T cells were linked to increased infection and mortality (11). Table 1 provides a list of conditions in which a role of CD4^+^CD28^−^ T cells was reported or investigated.

**Table 1:** **Conditions in which CD4^+^CD28^−^ T cells were reported and/or investigated**.

Cardiovascular (2, 3, 5, 12–17)	Autoimmune (5, 6, 9, 11, 18–25)	Others (26)
Angina pectoris	Rheumatoid arthritis	Renal transplant dysfunction
Acute coronary syndrome	Granulomatosis with polyangiitis	
Myocardial infarction	Diabetes	
Chronic heart failure	Systemic lupus erythematosus	
Abdominal aortic aneurysms	Multiple sclerosis	
	Ankylosing spondylitis	
	Crohn’s disease	
	Graves’ disease	
	Autoimmune myopathy	
	Dermatomyositis	
	Polymyositis	
	Polymyalgia rheumatica and giant cell arteritis	

## CMV Infection Triggers the Expansion of CD4^+^CD28^−^ T Cells

There is a striking link between CD4^+^CD28^−^ T cells and CMV infection. Work in renal transplantation has demonstrated that the emergence and expansion of CD4^+^CD28^−^ T cells in CMV-seronegative (CMV^−^) graft recipients directly results from infection by a CMV-seropositive (CMV^+^) graft. Recipients showed detectable levels of CD4^+^CD28^−^ T cells just after the clearance of CMV viral load, and the proliferation of these cells *in vitro* could be stimulated by CMV antigen but not tuberculin or tetanus toxoid, for example. However, CD4^+^CD28^−^ T cells did not emerge in CMV^−^ recipients of CMV^−^ grafts (27). Furthermore, CMV-specific CD4^+^ T cells are in large part CD28^−^ (28). Given that *ex vivo* T cell stimulation cannot adequately cover all CMV antigens, it has remained unclear if all CD4^+^CD28^−^ T cells are CMV-specific or if some of them expand after CMV infection for reasons yet to be discovered. Interestingly, Zal et al. reported that in patients with ACS and/or chronic stable angina CD4^+^CD28^−^ T cells (partially) responded to HSP60 but not to a CMV lysate (29). It is important to note, however, that CMV lysates (prepared from lytically CMV-infected human fibroblasts) are not an all-inclusive collection of CMV antigens (30). It is possible, therefore, that CD4^+^CD28^−^ T cells specific for antigens not represented in the lysate cross-reacted with HSP60. Cross-reactivity between HSP60 and the CMV UL122 and US28 proteins has indeed been described for antibodies, which might be an indirect mechanism by which CMV infection facilitates endothelial cell injury (31).

**Figure 1:**
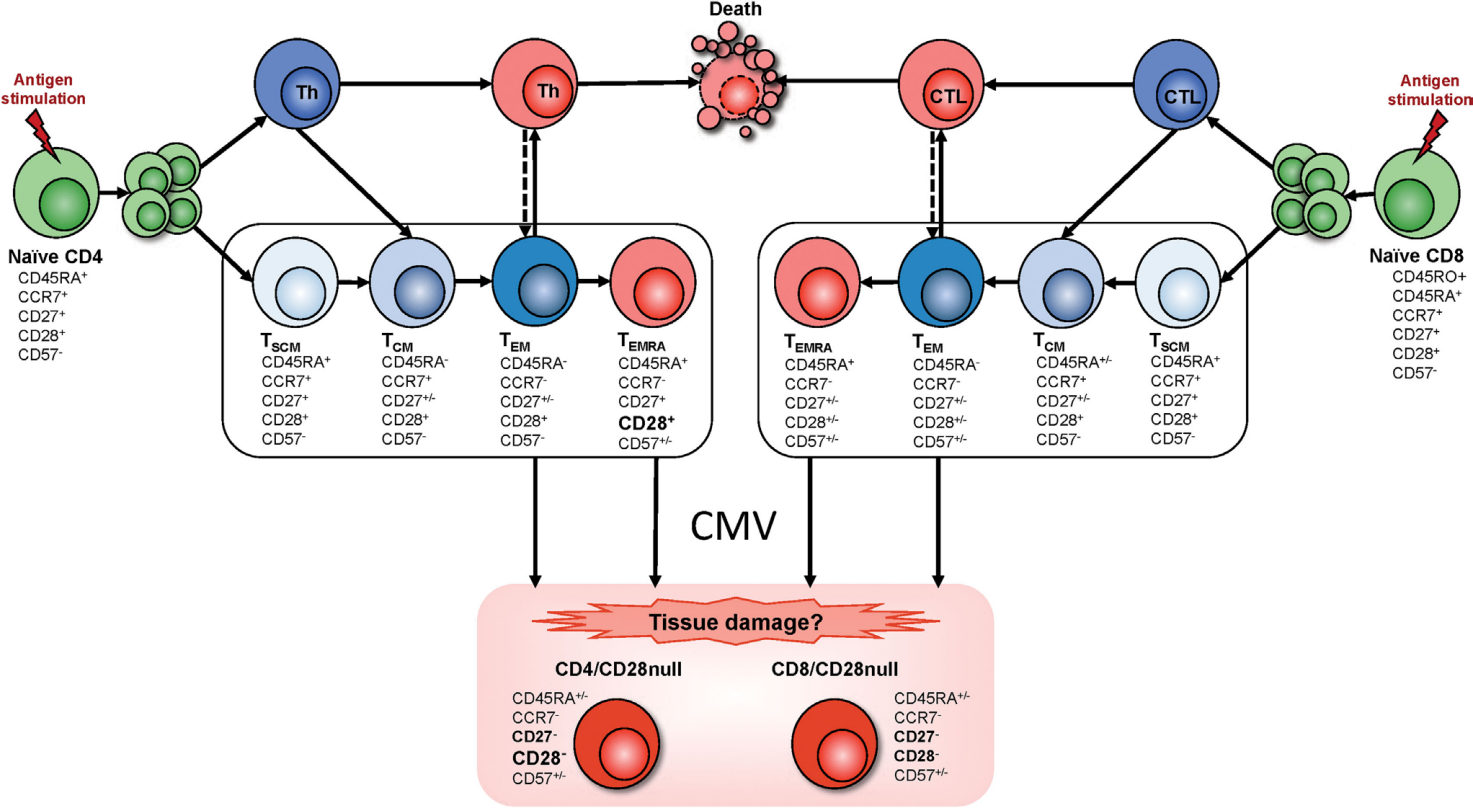
**T cell differentiation and the emergence of CMV-induced T cell phenotypes**. Memory T cell differentiation is regulated by intracellular and extracellular factors. Mechanisms of memory development upon naïve T cell activation (antigen stimulation) are the subject of ongoing discussion. Since it has been reported that CD4^+^ T cell memory development resembles that of CD8^+^ T cells (34), we assumed that both T cell subsets follow similar pathways. However, transitional memory subsets sitting between central memory T cells (T_CM_) and effector memory T cells (T_EM_) have been described in the CD4^+^ T cell compartment. Several memory T cell subsets have been defined but their lineage relationship has remained unclear. Some models describe a linear origin of memory T cells directly from effector T cells; other models propose a divergent differentiation where naïve T cells give rise to memory and effector T cells through asymmetrical division. More recently a progressive differentiation pathway has been proposed, depending on stimulus intensity and duration (represented inside the box). According to this model, T cell fate depends on the duration of signaling and presence/absence of cytokines. Brief stimulation leads to the generation of T_CM_ whereas sustained stimulation plus presence of cytokines generates T_EM_. Therefore, in the progressive model, a single naïve T cell will give rise to different memory T cell subsets that are the precursors of terminally differentiated effector T cells. Progression into these differentiated memory subsets relies on the gradual response to cytokines, acquisition of tissue homing receptors, resistance to apoptosis, and gain of effector functions while gradually losing lymph node homing receptors, proliferative capacity, and the ability to produce IL-2 production, to self-renew, and survive [for review, see Ref. (35–39)]. Although the exact origin of the CD28^−^ T cell phenotype is not clear, based on the literature, we hypothesize that these cells arise from terminally differentiated effector memory T cells (T_EMRA_) as well as T_EM_ after exposure to CMV. Abbreviations: Th, T-helper cell; CTL, cytotoxic T cell; T_SCM_, stem cell memory T cell; T_CM_, central memory T cell; T_EM_, effector memory T cell; T_EMRA_, terminally differentiated (CD45RA reexpressing) effector memory T cell.

Strikingly, not a single study has reported accumulations of CD4^+^CD28^−^ T cells in CMV-uninfected individuals; however, some studies have reported low frequencies of these cells in CMV^−^ people in the order of 1–2% of CD4 T cells (11). Of note, in the context of inflammatory diseases such as RA and GPA, CMV-driven expansions of CD4^+^CD28^−^ T cells are accentuated compared to otherwise healthy individuals, which will increase the potential for tissue damage (11, 32). Based on the literature, we have drafted a model of CMV antigen-driven T cell differentiation toward the emergence of CD4^+^CD28^−^ T cells (Figure 1). This pathway is different from pathways leading to T cell exhaustion, which are typically associated with a loss of effector functions (33).

## CD4^+^CD28^−^ T Cells are Terminally Differentiated Effector Cells

Before CD4^+^ T cells lose CD28 expression, they will have lost the expression of a number of other molecules, in particular the costimulatory receptor, CD27, and gained expression of memory markers (40). Unlike normal helper T cells, CD4^+^CD28^−^ T cells do not provide help to B cells; however, they express NK-cell receptors, in particular killer activating receptors (18, 19, 23). They produce more TNF-α and IFN-γ and are more cytotoxic than CD4^+^CD28^+^ T cells (17, 41). CD4^+^CD28^−^ T cells may home to atheromatous lesions because they express the chemokine receptors, CXCR3, CCR6, and CCR7 (17, 24). Of note, vascular EC are primary CMV infection targets (42). Synovial fluid CD4^+^CD28^−^ T cells from RA patients produce less IFN-γ and TNF-α than their circulating counterparts and, unlike them, also produce IL-17A (24). Additionally, they produce perforin and granzyme B, which can destroy synovial tissue (41, 43, 44). Reduced responsiveness to CD4^+^CD25^+^ regulatory T cells and resistance to apoptosis further add to their destructive potential (22, 45). Table 2 lists the most prominent features of CD4^+^CD28^−^ T cells.

**Table 2:** **Properties of CD4^+^CD28^−^ T cells**.

Molecule type/property	Specific molecules/properties identified (25, 46, 47)
Costimulatory receptors	CD27^−^, CD40L-, OX40^+^ (CD134), 4-1BB^+^ (CD137)
Chemokine receptors	CCR7^−^, CX3CR1^+^ (fractalkine receptor), CCR5^+^
Toll-like receptors	TLR2^+^, TLR4^+^
Natural killer receptors	KIR^+^, NKG2D^+^, CD11b^+^, CD161^+^, NKG2C^+^
Cadherin/integrin	VLA-4^+^, ICAM-1^+^
Cytokines and mediators	IFN-γ^+^, TNF-α^+^, IL-2^+^, perforin^+^, granzyme B^+^
Other features	– increased resistance to apoptosis – increased resistance Treg suppression – slow division rate (replicative senescence)

## CMV Involvement in Cardiovascular Disease (CVD)—Clinical Observations and Epidemiology

CMV infection has been associated with vascular pathology ever since the virus was isolated from atherosclerotic lesions, but it was unclear if it played a causative role (48). To date, there is strong epidemiologic evidence that CMV is the major driver of premature CVD in HIV-infected people (49) and increasing recognition of an association with higher CVD mortality in HIV-uninfected people (50). Meanwhile, a role for CMV in driving/accelerating autoimmune disease has been the subject of discussion since the early 1990s (51). Of particular interest to this review, several authors have shown that CMV infection exacerbates inflammation in RA (11, 52–54), with one study indicating that higher anti-CMV antibody levels associate with more frequent surgical procedures and more severe joint damage (53). Several authors have shown that in RA patients CMV antigens are indeed detectable in synovial tissue (55, 56). Also, high numbers of virus-specific T cells including CMV-specific T cells can be found at these sites (52). Table 3 shows CV and autoimmune conditions in which CMV has been implicated.

**Table 3:** **Cardiovascular and autoimmune conditions in which a role of CMV infection has been suspected or confirmed**.

CV (31, 57–60)	Autoimmune (11, 51, 61, 62)
Atherosclerosis	Rheumatoid arthritis
Hypertension	Lupus erythematosus
Coronary heart disease	Sjögren’s syndrome
	Granulomatosis with polyangiitis
	Diabetes mellitus
	Systemic sclerosis

There are several epidemiological links between CMV infection and CVD. In particular, lower socioeconomic position (SEP) correlates with a higher prevalence of dyslipidemia, higher cholesterol, and smoking, which are all risk factors for CVD. However, lower SEP is also associated with a high prevalence of CMV infection (63). Therefore, CVD and CMV are significantly correlated at an epidemiological level in such populations, which complicates the analysis. A recent cross sectional study, however, found that despite this complex interrelatedness of risk factors, CMV infection may explain partly the relationship between SEP and CVD (64). There is also epidemiological evidence that CMV is a driver of heart disease in HIV^+^ women (65). The complexity and importance of this issue was recently highlighted (49).

## Evidence Linking (CMV-Specific) T Cells to Hypertension, Vascular Pathology, and Acute Coronary Events

The evidence for a role of T cells in myocardial infarction has recently been reviewed identifying direct involvement of CD4^+^ and CD8^+^ T cells in both coronary artery injury and healing/remodeling with regulatory T cells being particularly involved in the latter (66).

**Figure 2:**
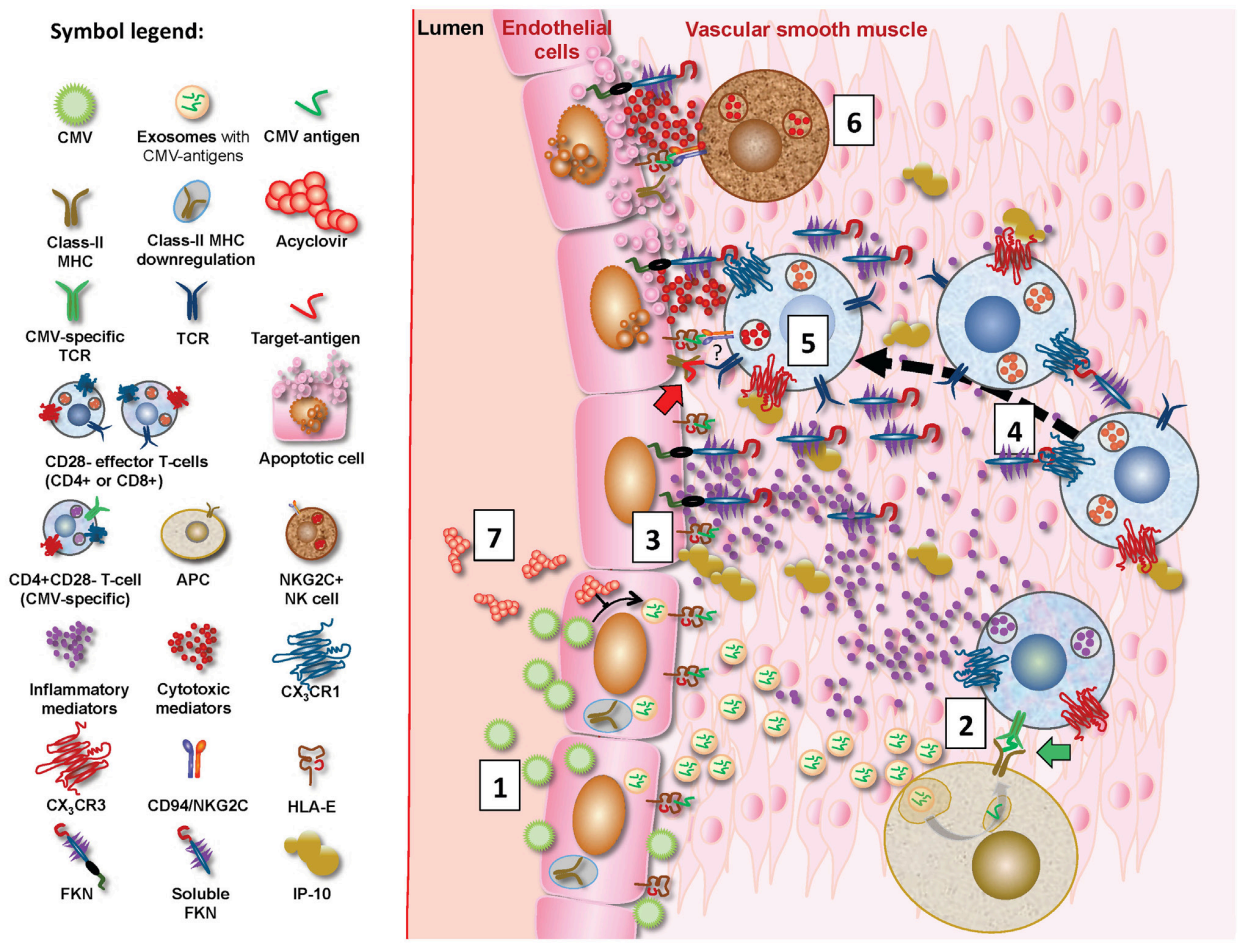
**Proposed mechanisms for CMV-driven vascular damage**. CMV-infected EC will downregulate MHC expression but produce non-infectious exosomes (NIE) loaded with CMV proteins, in particular UL55 (gB) (1) (68), allowing effective CMV antigen presentation by antigen-presenting cells (APC) following NIE uptake/processing. Vasculature-infiltrating CMV-specific CD4^+^ effector T cells will hence encounter these antigens on APC (shown as green CMV antigen in diagram; green block arrow) (2) and subsequently produce pro-inflammatory mediators such as IFN-γ. These induce the expression of fractalkine (FKN), IFN-γ-inducible protein 10, and possibly additional chemokines in EC (3) (69), which in turn attract infiltrating CD4^+^CD28^−^ and probably also CD8^+^CD28^−^ T cells to the ECs (4). These may be CMV-specific but possibly also non-CMV-specific (symbolized by red “target antigen” in diagram; red block arrow). They may kill ECs through perforin/granzyme secretion (5). Despite CMV infection, HLA-E expression remains unaffected in EC, so that interaction between HLA-E on EC and CD94/NKG2C on NK cells may also trigger CD94/NKG2C-mediated cytotoxicity (6) (71). NKG2C^+^ NK cells are known to be expanded by CMV infection and it is noteworthy that CD4^+^CD28^−^ T cells may also express NKG2C (indicated by “?” in diagram). Acyclovir reduces CMV-specific T cell responses by inhibiting replication (72) and will probably reduce NIE formation in infected EC, thus reducing antigen presentation by APCs and subsequent effector T cell activation (7).

Following CMV infection of EC, class-II MHC expression in these cells is reduced hampering CMV-antigen presentation to CD4^+^ T cells (67). However, CMV-infected EC can release non-infectious exosomes (NIE) that are replete with CMV proteins, in particular UL55, a major CD4^+^ T cell target protein. Uptake of NIE by APC leads to effective presentation of CMV antigens to CD4^+^ T cells (68). Moreover, pro-inflammatory mediators released by PBMCs in response to CMV can induce expression of fractalkine (FKN) and inducible protein 10 (IP-10) in EC. These specifically bind the chemokine receptors CX3CR1 and CX_3_CR3, respectively, which are expressed on effector CD4^+^ and CD8^+^ T cells in CMV-infected individuals (69). We hypothesize that vasculature-infiltrating CD4^+^CD28^−^ effector T cells expressing CX_3_CR1 and/or CX_3_CR3 are, therefore, attracted to FKN and IP-10-producing EC. Cytotoxic molecules secreted by CD28^−^ T cells (Table 2) may then trigger EC death by apoptosis. Of interest, CMV immune evasion includes downregulation of class-I MHC expression on infected EC but leaves HLA-E expression unaffected. NKG2C^+^-expressing NK cells and T cells expand in CMV infection, and NKG2C^+^-mediated cytotoxicity is triggered by the interaction between CD94/NKG2C and HLA-E molecules on CMV-infected EC (70, 71). Figure 2 provides a synopsis of these mechanisms.

Work in mouse models has also confirmed a role for T cells in hypertension, an important contributor to vascular damage; RAG-1 double-knockout (RAG-1^−^/^−^) mice lacking both T cells and B-cells showed blunted hypertension in response to angiotensin-II infusion or (DOCA)-salt. They also exhibited decreased vascular reactive oxygen species (ROS) production with reduced consumption of the relaxing factor, nitric oxide (NO). Adoptive transfer of T cells (but not B-cells) restored these effects to normal (73). Others showed that murine CMV (MCMV) infection leads to hypertension within weeks independently of atherosclerotic plaque formation, but at the same time contributes to (aortic) atherosclerosis, which might result from persistent CMV infection of EC inducing renin expression (74). This will in turn increase local angiotensin-II levels, which might activate angiotensin-II receptor positive infiltrating T cells to produce more ROS. Recently, Pachnio et al (75). have confirmed that CMV-induced CD4^+^CD28^−^ T cells indeed have all the necessary properties required to infiltrate the vasculature.

## RA and CV Complications

As a result of an excess of CV events, the life expectancy of RA patients is reduced by 3–10 years compared with the general population (76, 77). The risk of CVD-associated death is up to 50% higher in RA patients than controls, and the risks of ischemic heart and cerebrovascular diseases are elevated to a similar extent (78). RA is the most common inflammatory joint disease worldwide, affecting about 1% of the population (77). RA is characterized by infiltration of the synovial membranes by pro-inflammatory immune cells, swelling and deformity of joints and excess synovial fluid containing infiltrating immune cells and cytokines (79, 80). Extra-articular manifestations are widespread and involve the CV system (81).

Traditional CVD risk factors such as smoking, physical inactivity, hypertension, and DM contribute to death from CVD in RA but do not have the same predictive value as in patients without RA (77, 82). There is some evidence that RA itself accelerates atherogenesis (83). Also, following myocardial infarction patients with RA have considerably higher 30-day case fatality rates (76). Chronic inflammation is a normal consequence of aging (84) and a key player in atherogenesis. It promotes endothelial cell activation and vascular dysfunction and, together with other risk factors, leads to arterial wall thickening, promotes atheromatous changes, induces decreased vascular compliance, and contributes to increased blood pressure. This further promotes vascular damage in a self-perpetuating cycle. Ultimately, blockage of blood vessels may lead to myocardial infarction or stroke (76, 85).

## CD4^+^CD28^−^ T Cells Arise as an Obvious Mechanistic Link Between CMV Infection, CVD, and RA

The vast majority of studies investigating the presence and role of CD4^+^CD28^−^ T cells in CVD and autoimmune diseases did so without considering participant CMV infection status, suggesting that many researchers are unaware of the association of an expansion of this subset with CMV infection. The most relevant details from a number of such reports are found in Tables S1 and S2 in Supplementary Material. Only a handful of studies explored the presence of CD4^+^CD28^−^ and/or CD8^+^CD28^−^ T cells in CVD or autoimmune disease in the context of CMV infection status. Interestingly, most of these included CMV^+^participants only. We identified only two studies that included CMV^+^ and CMV^−^ participants (Table 4). Among the studies not accounting for CMV status, several reported significant differences between RA patients and healthy controls with respect to the frequency of CD4^+^CD28^−^ T cells (5, 8, 20, 32). Also, major differences were reported between cases with limited RA and extra-articular RA (21). On the whole, between 3 and 10 times, more CD4^+^CD28^−^ T cells were reported in RA compared to healthy controls. With respect to CVD, Liuzzo et al. found ninefold higher levels of CD4^+^CD28^−^ T cells in patients with unstable angina compared to those with stable angina; these differences were later confirmed in a second study (2, 3). Rizzello et al., by contrast, found “only” a 2.5-fold difference in CD4^+^CD28^−^ T cell levels between such groups (13). Others reported frequencies of CD4^+^CD28^−^ lymphocytes (rather than T cells) as a percentage of all lymphocytes, which makes their data difficult to compare (17).

**Table 4:** **CD4^+^CD28^−^ T cells in studies stratified by CMV status**.

Reference	Disease	Number of individuals in study	M:F ratio	Age range or *IQR* in years (*median*) and/or mean ± SD	Cell subset investigated	% of reference subset given as mean or *median* or absolute counts/µl blood mean ± SD	
						CMV^+^	CMV^–^
Thewissen et al. (22)	Rheumatoid arthritis (RA)	4	1:3	59–76 (67)	CD4^+^CD28^−^	9.6	n.k.
	HC	4	3:1	30–48 (35)		9.3	n.k.
Morgan et al. (11)	GPA^a^	48	25:23	47–74 (*64*)	CD4^+^CD28^−^	*19*	*0.8*
	HC	38	13:25	41–77 (*57)*		*22*	*1.4*
Pierer et al. (53)	RA	202	49:153	*51–68* (62)	CD4^+^CD28^−^	*8.15*	*0.37*
Jonasson et al. (86)	Cardiovascular disease	43^b^	All males	55.1 ± 5.6	CD4^+^CD28^−^	6.7	
					CD8^+^CD28^−^	452 ± 258	172 ± 174
					CD8^+^CD57^+^	392 ± 226	167 ± 183
	HC	69^b^	All males	49.5 ± 5.9	CD4^+^CD28^−^	5.8	
					CD8^+^CD28^−^	329 ± 216	112 ± 71
					CD8^+^CD57^+^	269 ± 190	105 ± 67

*n.k., not known; HC, healthy control*.

*^a^GPA, granulomatosis with polyangiitis, was used here as a comparative inflammatory disorder*.

*^b^67% of patients and 61% of controls were CMV^+^*.

*Significance of Italics: % of reference subset given as median, as indicated in the headings of the columns*.

Reports in GPA and RA patients clearly confirm that significant expansions of the CD4^+^CD28^−^ T cell subset only occur in CMV^+^ individuals. The levels of these cells were 24-fold higher and 22-fold higher in CMV^+^ compared with CMV^−^ GPA and RA patients, respectively (11, 53). Also, the relative expansions in CMV^+^ compared to CMV^−^ individuals were significantly accelerated in the presence of GPA as they were increased “only” by factor 14 higher in healthy controls. The remaining studies listed in Table 4 report CD4^+^CD28^−^ T cell frequencies in CMV^+^ individuals only.

In summary, the listed reports argue strongly in favor of a role of CMV infection in CV complications, most likely as a result of the distribution of the CD4^+^CD28^−^ subsets in the disease and control groups.

## Could CD4^+^CD28^−^ T Cells be Targeted by Immunotherapies?

Experimental evidence suggests that anti-CMV treatment could reduce the reactivity as well as the numbers of CMV-specific T cells. Particularly, low dose acyclovir therapy decreases the CD4^+^ T cell response to pp65 CMV protein, most likely by diminishing the CMV-antigen load, turnover, and uptake by APC (72). In addition, there is evidence from mouse models that, at least in older mice, valacyclovir treatment leads to an 80% reduction of the CD8^+^ T cell response to MCMV (87). If CMV-specific T cells were actually involved in mediating CMV-driven vascular damage, then a possible approach to slow down this process would be the use of anti-viral drugs.

Therapies based on the direct targeting of CD4^+^CD28^−^ T cells have been investigated in several conditions. To this regard, the effects of different therapeutic regimens on CD4^+^CD28^−^ T cell frequencies have been investigated in patients with hyperinsulinemic polycystic ovary syndrome, in which increased frequencies of this subset have also been observed (but an association with CMV has not been investigated). Treatment with drospirenone–ethinylestradiol and metformin resulted in a significant reduction of frequencies of CD4^+^CD28^−^ T cells (88). Moreover, it has been demonstrated in organ transplant recipients that treatment with polyclonal anti-thymocyte globulin preferentially triggers apoptosis in CD4^+^CD28^−^ compared to CD4^+^CD28^+^ T cells (89). Other therapies targeting the functional capacity of these cytotoxic cells have been investigated as well. The only K^+^ channels present in CD4^+^CD28^−^ T cells from ACS patients are Kv1.3 and IKCa1. Blockade of the Kv1.3 channel by 5-(4-phenoxybutoxy)psoralen (PAP-1) resulted in suppression of the pro-inflammatory function of CD4^+^CD28^−^ T cells (90), however, did not appear to induce general immunosuppression. In a rat model, chronic administration of PAP-1 prevented the development of unstable atherosclerotic plaques, most probably by blocking the release of inflammatory and cytotoxic molecules from CD4^+^CD28^−^ T cells (91). Finally, in RA patients treated with abatacept, a reduction of circulating CD4^+^CD28^−^ T cells has been observed, and it was correlated with a reduction of disease activity (92–94). Similar results were observed by Pierer et al. (95) in RA patients treated with TNF-α blocking agents (etanercept and infliximab). Anti-TNF therapy has been shown to diminish the myocardial infarction risk and to increase vascular compliance (96, 97). At the same time, it reduces the number of CD4^+^CD28^−^ T cells (13). However, little is known about how other drugs used in RA affect CV complications (recently reviewed in this journal) (98).

## Conclusion

We believe that the literature reviewed in this article explains to a large extent the striking epidemiological association reported between CMV infection and increased CV mortality (50, 60, 99–102). It is, in particular, the emerging, immediate and specific role of CD4^+^CD28^−^ T cells in both acute and chronic vascular pathology that takes this association to a higher level. This is, because expansion of this T cell subset beyond a very small percentage (1–2% of CD4^+^ T cells) is *exclusively* found in CMV^+^ individuals. Literature from the fields of chronic inflammation/autoimmunity, CVD, and viral immunology, together provide a fascinating insight into the effects of expanded populations of cytotoxic, CD4^+^CD28^−^ T cells. These are ultimately driven by a common virus infection, whose burden on the immune system is still being underestimated (103).

## Author Contributions

IB: literature search, review, compilation of supplementary tables including all relevant information, and initial draft. AP: literature search, review, revising, and rewriting draft, figures’ design and drawing, and writing/editing final manuscript. GM: literature review and contributions to writing and editing final manuscript. KD: contributions to writing manuscript, editing, and contribution to tables and figures. FK: initiation of the work, literature review and selection, contributing to tables and figures, critical review of all information, and writing of the final manuscript.

## Conflict of Interest Statement

The authors declare that the research was conducted in the absence of any commercial or financial relationships that could be construed as a potential conflict of interest.
