# Supplementary material for: Expansions of Cytotoxic CD4+CD28− T Cells Drive Excess Cardiovascular Mortality in Rheumatoid Arthritis and Other Chronic Inflammatory Conditions and Are Triggered by CMV Infection
Source: Front Immunol. 2017 Mar 2;8:195. doi: 10.3389/fimmu.2017.00195 (PMC5332470; doi:10.3389/fimmu.2017.00195)
Supplement: Supplementary file 1 [file Table_1.DOCX]

**Broadley et al. , Supplementary Table 1**

**Supplementary Table 1: CD4+CD28− T cells in cardiovascular disease**

| **Study (year) [ref]** | **Type of study** | **Disease** | **Number of individuals in study** | **M:F ratio** | **Age range or *IQR* in years (*median*) and/or mean +/- STD** | **Cell Subset**  **investigated** | **% of reference subset given as mean or *median*** |  |
| --- | --- | --- | --- | --- | --- | --- | --- | --- |
| *Liuzzo (1999) (Liuzzo et al., 1999)* | Cohort | UA^a^ | 25 | 16:9 | 67 +/- 10 | CD4+CD28− | *9* |  |
|  |  | SA^b^ | 25 | 21:4 | 64 +/- 10 |  | *p<0.001* |  |
| *Liuzzo (2000) (Liuzzo et al., 2000)* | Cross-Sectional | UA^a^ | 34 | 20:14 | 66 +/- 11 | CD4+CD28− | *10.8* |  |
|  |  | SA^b^ | 34 | 27:7 | 64 +/- 11 |  | *1.5* |  |
| *Rizzello (2006)(Rizzello et al., 2006)* | longitudinal | UA^a^ | 17 | 9:8 | 67 +/- 8 | CD4+CD28− | *6.2* |  |
|  |  | UA^c^ |  |  |  |  | *4.1*^c^ |  |
| *Brugaletta (2006) (Brugaletta et al., 2006)* | Cohort | UA^a^ | 78 | 53:25 | 65 +/- 9 | CD4+CD28− | *3* |  |
|  |  | UA Statins^d^ | 33 | 26:7 | 62 +/- 9 |  | *2.3* |  |
| *Alber (2009) (Alber et al., 2009)* | Cross-Sectional | SA^b^ | 30 | all male | 38-68  55.4 +/- 7.4 | CD4+CD28− | 2.6 |  |
|  |  |  |  |  |  | CD8+CD28− | 31.1 |  |
| *Koller (2013) (Koller et al., 2013)* | Cohort | Chronic Heart Failure | 107 | 83:24 | 57.2-70.6 (65.7) | CD4+CD28− | *57* had frequency of ≥3.4^e^ |  |
|  |  |  |  |  |  |  | *23* had frequency between 4-10^e^ |  |
|  |  |  |  |  |  |  | *27* had frequency of >10^e^ |  |
| *Teo (2013) (Teo et al., 2013)* | Cross-Sectional | HC^f^ | 16 | 10:6 | 40-80  58 +/-5.2 | CD4+CD28− | 0.5^i^ |  |
|  |  | RF^h^ | 22 | 8:14 | 40-80  57.8 ± 7 |  | 0.65^i^ |  |
|  |  | SA^b^ | 30 | 25:5 | 40-80  61.6 +/- 7.1 |  | 0.7^i^ |  |
|  |  | ACS^g^ | 20 | 12:8 | 40-80  62.1 +/- 12.6 |  | 0.6^i^ |  |
| *Tae Yu (2014) (Tae Yu et al., 2014)* | Cohort | MI^j^ | 55 | 33:15^k^ | 65 + /- 13 | CD8+CD28− | 24.3 |  |
|  |  |  |  |  |  | CD4+CD28− | 3.1 |  |
|  |  |  |  | 6:1^l^ | 74 + / - 8 | CD8+CD28− | 42.3 |  |
|  |  |  |  |  |  | CD4+CD28− | *3.6* |  |

^a^UA Unstable angina; ^b^SA Stable angina; ^c^UA Patients were treated under twenty four hours incubation of blood with *100*µ*g/mL* of infliximab; ^d^UA Statins Patients, treated with statins 4 weeks prior to investigation (79%) took 20 mg/day atorvastatin, four patients (12%) took simvastatin 20 mg/day, and the remaining three patients (9%) took pravastatin 10 mg/day; ^e^Patients % of CD4+CD28− T-cell subsets was recorded within 3 different ranges. The numbers of patients who had these ranges of frequency were also recorded. The absolute minimum average which could have been recorded is given as 3.4%; ^f^HC Healthy control; ^g^ACS Acute coronary syndrome; ^h^RF Risk factor group included patients with at least one risk factor for atherosclerosis (hypertension, diabetes, dyslipidemia or smoking); ^i^Estimates taken from graph. ^j^Myocardial infarction; ^k^Patients who survived short-term MI; ^l^Patients with cardiovascular mortality.
