# Supplementary material for: Expansions of Cytotoxic CD4+CD28− T Cells Drive Excess Cardiovascular Mortality in Rheumatoid Arthritis and Other Chronic Inflammatory Conditions and Are Triggered by CMV Infection
Source: Front Immunol. 2017 Mar 2;8:195. doi: 10.3389/fimmu.2017.00195 (PMC5332470; doi:10.3389/fimmu.2017.00195)
Supplement: Supplementary file 2 [file Table_2.DOCX]

**Broadley et al. , Supplementary Table 2**

**Supplementary Table 2: CD4+CD28− T cells in arthritis and autoimmune disease**

| **Study (year) [ref]** | **Disease** | **individuals in study (n)** | **M:F ratio** | **Age range or *IQR* in years (*median*) and/or mean +/- STD** | **Cell subset investigated** | **% of reference subset, result given as mean or *median*** |
| --- | --- | --- | --- | --- | --- | --- |
| *Schmidt (1996) (Schmidt et al., 1996a)* | RA^a^ | 22 | n.k. | Age matched | CD4+CD28− | *9.7* |
|  | HC^b^ | 22 | n.k. | Age matched |  | *1.7* |
| *Namekawa (1998) (Namekawa et al., 1998)* | RA | 6 | n.k. | n.k. | CD4+CD28− | 5.90^c^ |
| *Namekawa (2000) (Namekawa et al., 2000)* | RA | 10 | n.k. | n.k. | CD4+CD28− | 2.3-19.4^d^ |
| *Warrington (2001) (Warrington et al., 2001)* | RA | 36 | 16:20 | 57 +/-12 | NK receptor^e^ | *15.8* |
|  | HC | 40 | 1:1 | 51 +/-11 |  | *12.5* |
| *Komocsi (2002) (Komocsi et al., 2002)* | GPA^f^ | 12 | 1:1 | 53.4 +/- 3 | CD4+CD28− | 14.4 |
|  |  |  |  |  | CD8+CD28− | 40.8 |
|  | HC | n.k. | n.k. | n.k. | n.k. | n.k. |
| *Gerli (2004) (Gerli et al., 2004)* | RA | 87 | 25:62 | 63 +/-10 | CD4+CD28− | *8.3* |
|  | HC | 33 | 9:24 | 64.2 +/-10 |  | *2.7* |
| *Bryl (2005) (Bryl et al., 2005)* | RA | 26 | 1:3 | 18-88 (*55*) | CD4+CD28− | 10^g^ |
|  |  |  |  |  | CD8+CD28− | 45^g^ |
|  | HC | 17 | 1:2 | 21-85 (*58*) | CD4+CD28− | 1^g^ |
|  |  |  |  |  | CD8+CD28− | 30^g^ |
| *Michel (2007) (Michel et al., 2007)* | Extra-articular RA | 45 | 11:34 | 63.9 +/-11.5 | CD4+CD28− | 22.67 |
|  |  |  |  |  | CD8+CD28− | 45.28 |
|  | limited RA | 27 | 1:2 | 60.7 +/-13.6 | CD4+CD28− | 6.9 |
|  |  |  |  |  | CD8+CD28− | 31.9 |
| *Thewissen (2007) (Thewissen et al., 2007b)* | RA | 57 | 18:37 | 34-84 (56.3) | CD4+CD28− | 4.99 |
|  | HC | 59 | 21:38 | 20-85 (43.5) |  | 1.78 |
| *Fasth (2010) (Fasth et al., 2010)* | RA | 7 | 3:4 | 52-79 (62.9) | CD4+CD28− | 17.8 |
| *Pieper (2014) (Pieper et al., 2014)* | RA | 44 | 1:3 | 18-86 (57) | CD4+CD28− | ≥5^h^ |

^a^RA Rheumatoid arthritis; ^b^HC Healthy control; n.k. data not known; ^c^5.90 Mean was estimated from 6 results, two of which were given as <1.0. In this case 0.99 was used as this would be the largest result possible for the two means; ^d^2.3-19.4 a mean range was given for this study; ^e^NK receptor expression on CD4+CD28− T Cells was used as a comparative inflammatory cell subset biomarker; ^f^GPA Granulomatosis with Polyangiitis (Wegner’s) was used here as a comparative inflammatory disorder; ^g^10 data recorded as a rough estimate taken from a graph; ^h^5 the mean given was equal to or >5%, therefore the lowest value for this mean can only be 5 which is presented here.
